# Correlation between the Amount of Anti-D Antibodies and IgG Subclasses with Severity of Haemolytic Disease of Foetus and Newborn

**DOI:** 10.3889/oamjms.2015.058

**Published:** 2015-05-30

**Authors:** Emilija Velkova

**Affiliations:** Institute of Transfusion Medicine in Republic of Macedonia, Vodnjanska 17, 1109 Skopje, Republic of Macedonia

**Keywords:** allosensibilisation, alloantibody, haemolitic desease of the foetus and newborn (HDFN), immunization, red blood cells, antenatal – postnatal, profilaxis, IgIG (hyperimun gamaglobulin)

## Abstract

**AIM:**

The aim of this study was to investigate the influence of subclasses to IgG anti-D on the intensity of hemolytic disease of fetus and newborn (HDFN) at 45 fetuses/newborns with symptoms of mild and severe HDFN in Republic of Macedonia.

**MATERIAL AND METHODS:**

In retrospective and prospective studies, in a period of 10 years, from 2004 to 2014, there have been immunohemathology tests performed on 22 009 samples on serums of pregnant women.

**RESULTS:**

At 37.78% of the total number of tested patients, IgG1 and IgG3 was the reason for severe HDFN. At 17.77% of the total number of tested patients, which had only IgG1detected, was the reason for serious intensity of HDFN. The correlation of the titer to anti-D antibodies in the mother’s serum and the intensity of HDFN were researched in 48 newborns. The titers between 1:8 and 1:32 resulted in 3 cases of HDFN with symptoms of severe disease and in 4 cases there were no signs of HDFN. At 12 women that had a titre between 1:32 and 1:512, five of the newborns developed severe HDFN, and seven had symptoms of mild and weak intensity form. In 3 cases the titer was higher than 512, and out of them one newborn had weak symptoms of HDFN, one developed severe HDFN and one ended with foetal death. Only in one case the titer reached a value higher than 1000, and it ended with a fetal death.

**CONCLUSIONS:**

The titers of the pregnant women serum those are lower than 32 and those higher than 1000 can well predict HDFN. The titers of anti-D antibodies between 64 and 512 have no exact predictive value. IgG1 and IgG3 subclasses of anti-D have no predictive value by themselves, and cannot foresee the outcome of HDFN. The research study results suggest that IgG1 and IgG3 should be included in a multi – parameter protocol for evaluation of the HDFN intensity. They can give a real assessment of the expected HDFN intensity in combination with the titer hight and the significance of the antibodies.

## Introduction

The Rh blood group system contains more than 40 antigens with extreme antigenicity. According to its importance regarding immune hematology it stands right next to the ABO system. It belongs to the group of trans-membrane proteins.

Basically, three Rh epitopes are defined within it, the Rh_o_ or the D antigen, which can be present or not, denoting Rh positive (D+), or Rh negative (D- or d) phenotype.

The Rh locus is assigned to the chromosome 1 (1p34.3-p36.13), it has two genes nearby the Rh locus that are responsible for the Rh protein, *RHD* и *RHCE* [1] D-positive individuals inherit both gens *RHD* and *RHCE*, while D-negative individuals inherit one *RHCE* gen from each parent. The deployment of the *RHCE* gene is used in general sense in order to mark the Rh gen which does not succeed to produce an antigen D.

Four common genes produce different combinations out of C, E, c and e: *RHCE, RHCe, RHcE, and RHce*. Besides the Rh gens, another gen which relates to RHAG (Rh – associated glycoprotein) is located in the chromosome 6 (6p11-p21.1), and it is connected to the Rh protein on the level of the red blood cell membrane [2].

The Rh genes act as autosomous co dominant alleles. The genetic material that codes C/c and E/e is tightly linked to one that controls D, that’s why the RH locus is considered as a unique gen complex during calculation of gen frequency.

**Figure 1:**
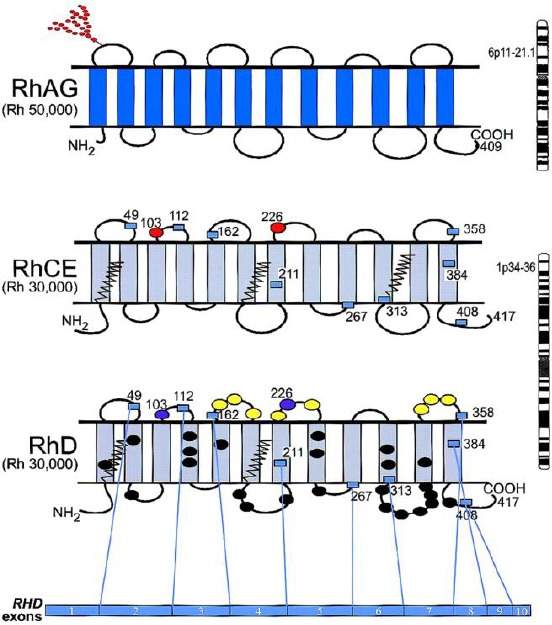
*Scheme of the Rh gen*.

Taking into consideration the available Rh antiserums (anti-D, anti-C, anti-c, anti-E, anti-e), the Rh phenotype of the tested red blood cells can be serologically defined. Accordingly, based on the phenotype and gen frequency regarding the tested population, the most probable Rh genotype can be estimated.

**Table 1:** Frequency of common Rh genotypes

Haplotype	Rh gens	White population	Black population	Americans	Asian
R_1_	RHD, RHCEe	0.42	0.17	0.44	0.70
R	RHce		0.37	0.26	0.11
R_2_	RHD, RHcE	0.14	0.11	0.34	0.21
R_0_	RHD, RHce	0.04	0.44	0.02	0.03
r’	RHCe		0.02	0.02	0.02
R’’	RHcE		0.01	0.00	0.06
R_z_	RHD, RHCE	0.00	0.00	0.06	0.01
r^y^	RHCE		0.00	0.00	0.00

Still, family research is the best way of establishing the right phenotype.

Exposure of D-negative individuals to D-positive red blood cells through transfusion or pregnancy, most probably will provoke an immune response. Transfusion of approximately 250 ml of D-positive red blood cells causes production of anti-D at about 80% of accidental D-negative recipients [3]. A significantly lower amount of red blood cells (approximately 1ml) will also stimulate production of anti-D at 50% of the D-negative recipients.

Most Rh antibodies are IgG, and usually IgG1, IgG3, or a combination of these two subclasses. The production of Rh antibodies is most often caused by transfusion or pregnancy.

The Rh antibodies are clinically significant and cause post-transfusion reactions and HDFN. The Rh antibodies, since they are primarily IgG1 and IgG3, cross through the placenta and can cause HDFN of various intensity. HDFN is directly dependant on the development of the red blood cell antigens on the surface of the fetal red blood cells. The D antigen can be detected in fetal red blood cells in the 5 to 6 gestation week [4, 5]. The antibodies persist for a longer period, 6 – 38 years [5].

HDFN is a clinical syndrome in which the basic pathophysiology disorder is the haemolytic anaemia of the fetus/newborn. HDFN starts during intrauterine life, and the reason for the disease is appearance of IgG antibodies that occur as a result of the mother’s alloimmunisation towards red blood cell antigens of the foetus that are inherited from the father, and are not present in the mother [6].

The application of RhD immune globulin prophylaxis up till 72 hours after giving birth, decreases the percentage of RhD sensibilisation to 1.5% or 2%, and the application of antenatal prophylaxis even lowered this percentage from 0.5% to 0.1% [7].

The present situation of application of antenatal, even postnatal prophylaxis in Republic of Macedonia is partial and incomplete. It does not comprise all pregnant women that are at risk of RhD sensibilisation. Unofficial data display that the prevalence of RhD sensibilisation in Republic of Macedonia is twice higher compared to the rest of the countries in Europe and the world, and it moves from 0,1% to maximum 2% [8-10].

The aim of this study was to investigate the influence of subclasses to IgG anti-D on the intensity of HDFN at 45 fetuses/newborns with symptoms of mild and severe HDFN in Republic of Macedonia.

## Material and Methods

In retrospective and prospective studies, in a period of 10 years, from 2004 to 2014, there have been immune hematology tests performed on 22,009 samples on serums of pregnant women: ABO, Rh blood group phenotypization; screening of anti red blood cell alloantibodies during pregnancy and after delivery with indirect antiglobuline test with five panels of red blood cell antigens, enzyme test with two panels of red blood cell antigens; direct antiglobuline test and elution of antibodies of the newborn red blood cells; identification of anti red blood cell antibodies with IAT and enzyme panel; quantification of antibodies with the titer method; blood group phenotypization of adequate antigens towards which antibodies were detected, regarding the mother, biological father and the newborn. All methods were performed according the producers recommendation and the Technical Manual, 14^th^ ed., American Association of Blood Banks [11].

Anti red blood cell antibodies were detected towards the antigens of the Rh system at 205 pregnant women, by identification of a total of 237 anti red blood cell antibodies. Out of them, only 169 had identified sensibilisation only towards the RhD antigen, and 36 of them had a presence of multiply antibodies.

**Table 2:** A risk to hemolysis according concentration of IgG subclasses

	IgG1		IgG2		control	IgG
Dilution	1 : 1	1 : 100	1 : 1	1 : 100		1 : 10
Reaction	2+	/	/	/	/	3+
Interpretation	Mild risk (IgG1 low concentration)					
Reaction	3+	2+	/	/	/	3+
Interpretation	High risk (IgG high concentration)					
Reaction	3+	2+	2+	/	/	3+
Interpretation	High risk (IgG high concentration, IgG3 low concentration)					
Reaction	/	/	3+	2+	/	3+
Interpretation	High risk (IgG3 high concentration)					
Reaction	2+	/	3+	2+	/	3+
Interpretation	High risk (IgG1 low concentration, IgG3 high concentration)					
Reaction	/	/	2+	/	/	3+
Interpretation	Mild risk (IgG3 low concentration)					
Reaction	3+	2+	3+	2+	/	3+
Interpretation	High risk (IgG1 И IgG3 high concentration)					

The study researched the influence of subclasses to IgG anti-D on the intensity of HDFN at 45 fetuses/newborns with symptoms of mild and severe HDFN.

In order to determine the degree of risk to hemolytic, which depends on the number of IgG1 and/or IgG3 molecules on the surface of red blood cells, as well as/or the presence of a complement, the test was performed by ID-cards DAT IgG1/IgG3. The gel contained two added dilutions of anti IgG1 and IgG3. The positive reaction with the first dilution matches coated red blood cells of 1000 IgG1 molecules, i.e. 125 IgG3 molecules per red blood cell. The positive results with the second dilution points out to high concentration of IgG1 and/or IgG3 antibodies.

## Results

IgG1 antibodies were detected in 15 samples of serums of sensibilised pregnant women towards the D antigen. Out of them, 7 had high concentration of IgG1, dilution 1:100. The tests performed on newborns came up with adequate results, i.e. symptoms of severe hemolytic disease. The rest of the 8 newborns demonstrated symptoms of mild HDFN or weak symptoms that needed no therapy. They have displayed low concentration of IgG1 that matches the dilution 1:1.

IgG1 together with IgG3 was detected at 25 pregnant women. At 10 of them the concentration to at least one of the subclasses was high, and at 6 of them there was a high concentration to both subclasses, received dilution 1:100. At all 16 newborns the performed tests from the umbilical cord showed adequate high concentration as in their mothers, with symptoms of severe HDFN. At 9 women both subclasses showed low concentration, and the newborns developed mild HDFN.

In 3 cases where only IgG3 anti-D antibody was detected, the newborns developed clinical picture of HDFN with a weak or mild intensity. There was not even one case of severe HDFN.

**Table 3:** Correlation of the type and amount of IgG anti-D subclasses with the HDFN intensity

IgG subclass	HDFN Total	HDFN weak/mild	HDFN severe	Dead fetus
IgG1	16	8	7	1
IgG1+ IgG3	26	9	16	1
IgG3	3	3	/	

In two cases the pregnancy ended with a dead fetus. The first one in the 21 gestation week and it had IgG1 and IgG3 detected in merely high concentration, and the second case ended in the 25 gestation week with also very high concentrations.

**Figure 2:**
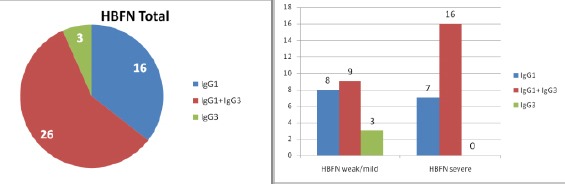
*Distribution of total HDFN (HBFN) according the IgG subclasses (left) and according the severity (right)*.

At all 205 sensibilised pregnant women we have performed titer tests to antibodies during the whole pregnancy, within intervals of 2 to 4 weeks depending on the significance of the antibody received from the results.

The correlation of the titer to anti-D antibodies in the mother’s serum and the intensity of HDFN were researched in 48 newborns.

The titers between 1:8 and 1:32 resulted in most cases with weak and mild form of HDFN. Only in 3 cases there was HDFN with symptoms of severe disease. In 4 cases there were no signs of HDFN.

At 12 women that had a titer between 1:32 and 1:512, five of the newborns developed severe HDFN, and seven had symptoms of mild and weak intensity form.

**Table 4:** Correlation of the titer of anti-D antibodies with the HDFN intensity

Titer	No symptoms or weak HDFN	Mild HDFN	Severe HDFN	Total
8-32	8	21	3	32

32-512	2	5	5	12

>512	1		2	3

>1000			1	1

In 3 cases the titer was higher than 512, and out of them one newborn had weak symptoms of HDFN, one developed severe HDFN and one ended with fetal death. Only in one case the titer reached a value higher than 1000, and it ended with a fetal death.

**Figure 3:**
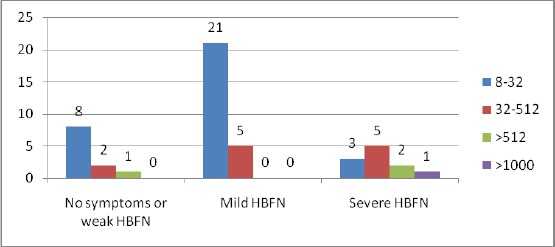
*Distribuition of the levels of anti-D antibodies according the severiti of HDFN (HBFN)*.

At titers lower than 32, the test was repeated every 4 weeks till the 24 gestation week, and after that every 2 weeks, with recommendation of obstetrics monitoring.

At titers higher than 32, the patients were immediately sent to intensive obstetrics monitoring. Depending on the results the titer was performed every 2 to 3 weeks, or immediately after performed invasive obstetrics procedure.

## Discussion

If the mother’s alloimmunisation is diagnosed, and the biological father of the fetus is well known, phenotypisation of the father is necessary for detection of antigen presence towards which the mother’s antibodies are focused.

If the father is antigen negative there is no possibility of the fetus to be antigen positive, and that’s why there is no risk of HDFN that could be caused by the mother’s alloantibodies.

When the father is heterozygote antigen positive, it is possible nowadays, in cases of D, C, c, E and K antibodies, to determine the antigen status of the foetus by a DNA test through the mother’s plasma. In cases when the mother is sensibilised, and the result of the foetal genotypisation is negative, we avoid all further analysis and multiple monitoring procedures during pregnancy.

If the result of the fetal genotypisation is positive, there is a necessity of immunohematology tests on every 2-4 weeks, by determining the type and amount of subclasses, and quantification of antibodies in general, as well as more detailed obstetrics tests related to risky pregnancy.

There are several classes of antibodies. Regarding allosensibilisation during pregnancy only two classes are of interest, IgM and IgG. If the antibody is identified as IgM, it means that it cannot find the placenta, thus cannot cause HDFN.

IgG is immunoglobulin that is most plentiful in the human serum. The subclasses differ in the constant region, especially in the structure of the upper CH2 domain. These regions are included in the bonding of the two receptors IgG-Fc (FcyR) I C1q. As a result, the subclasses have different effective functions in the sense of a trigger to FcyR – expessing cells, which produce phagocytosis or antibody – dependant cell cytotoxicity, and activation of the complement. There is an epytope on the Fc - receptor for neonatal Fc – receptor (FcRn), responsible for prolongation of the half life, placenta transport and IgG transport both ways through the mucosa surface [12].

IgG3 reacts more efficiently with the Fc – receptors on the macrophagys than the IgG1, but there is not enough data on in vivo destruction of red blood cells by IgG1 compared to IgG3 antibody [13]. IgG4 antibodies do not cause destruction of red blood cells since they have no capacity to activate a complement, neither can they interact with the macrophages. IgG2 is most often associated with the anti-A and anti-B antibodies. These antibodies can hardly cross the placenta and are less efficient mediators of macrophagys – induced clearance of red blood cells [13]. The serums containing anti-D antibodies most often reveal detection of IgG1 and IgG3, or just IgG1 or IgG3 subclass. The lytic activity of IgG3 is bigger than the one of IgG1 [12, 13].

According research data, IgG1 as a separate unit has a significantly wider specter of intensity compared to the IgG3, which is associated with a weak or mild HDFN, in concordance with scientific literature. IgG1 was detected in all three categories of HDFN, its distribution was significantly higher in cases of severe HDFN (p < 0.01).

At 37.78% of the total number of tested patients, and at 65.38% of the samples which had two antibodies detected, IgG1 and IgG3 was the reason for severe HDFN. At 17.77% of the total number of tested patients, and at 50% of the samples which had only IgG1detected, was the reason for serious intensity of HDFN.

Not a single patient with IgG3 had symptoms of severe HDFN. When both subclasses are present IgG1 and IgG3, HDFN has a serious intensity compared to the presence of only IgG1 or IgG3 alone. IgG3 is more associated to mild and weak form of HDFN.

The quantification of antibodies is usually performed by the titer method. The titer of the mother’s antibodies is the first step in evaluating RhD sensibilisation. A critical titer is defined as a titer associated to a significant risk of fetal hydrops [14].

The scientific literature gives different critical titers, from 8 to 32 for anti-D antibodies. The serum is recommended to be frozen and reestablished with the next sample by the same method.

The trend of subsequent levels of antibodies together with the previous obstetrics anamnesis is considered to be more important than any isolated result in prediction of the disease’ intensity [15-17]. In the study paper the intensity of HDFN in most cases responded to the titer hight to the anti-D antibody. This is particularly true for titers higher than 512. In certain cases where the antibody titer was above the critical point, and the newborn didn’t show signs of HDFN with a serious intensity as expected, determination of subclasses complied to a low concentration of IgG1 and/or IgG3, or the mother’s serum contained other subclasses of IgG that have no capacity of complement activation, or are less efficient mediators of macrophagys -induced clearance of red blood cells.

In conclusion, the titers of the pregnant women serum that is lower than 32 and those higher than 1000 can well predict HDFN. In the first example there is a rare occurrence of severe HDFN, and they are quite often manifested with no or very insignificant symptomatology. In the second case, over 1000, the occurrence of severe HDFN is inevitable. The titers of anti-D antibodies between 64 and 512 have no exact predictive value. IgG1 and IgG3 subclasses of anti-D have no predictive value by themselves, and cannot foresee the outcome of HDFN. The research study results suggest that IgG1 and IgG3 should be included in a multi – parameter protocol for evaluation of the HDFN intensity. They can give a real assessment of the expected HDFN intensity in combination with the titer hight and the significance of the antibodies.
